# Impaired energetics and normal myocardial lipids in rheumatoid arthritis and systemic lupus erythematosus: a phosphorous and proton magnetic resonance spectroscopy and cardiovascular magnetic resonance study

**DOI:** 10.1186/1532-429X-17-S1-O99

**Published:** 2015-02-03

**Authors:** Ntobeko A Ntusi, Cameron Holloway, Jane M Francis, Anne Davis, Eylem Levelt, Stefan K Piechnik, Vanessa M Ferreira, Paul M Matthews, Paul B Wordsworth, Theodoros D Karamitsos, Stefan Neubauer

**Affiliations:** 1 OCMR, Division of Cardiovascular Medicine, University of Oxford, Oxford, UK; 2 Division of Cardiology, Department of Medicine, University of Cape Town, Cape Town, South Africa; 3 Division of Brain Sciences, Imperial College London, London, UK; 4 Bortnar Institute, Nuffield Department of Orthopaedics, Rheumatology and Musculoskeletal Sciences, University of Oxford, Oxford, South Africa; 5 Department of Cardiology, University of New South Wales, Sydney, NSW, Australia

## Background

Rheumatoid arthritis (RA) and systemic lupus erythematosus (SLE) commonly involve the cardiovascular system and are associated with significant morbidity and mortality, driven by cardiovascular inflammation, microvascular and diastolic dysfunction and fibrosis. Cardiovascular magnetic resonance (CMR) can assess non-invasively cardiac function, strain, ischaemia, altered vascular function, perfusion, inflammation and fibrosis; magnetic resonance spectroscopy (MRS) provides further insights into the status of myocardial energetics and lipidosis. To date, there have been no cardiovascular MRS studies in RA and SLE patients. We hypothesised that RA and SLE would be associated with impaired myocardial energetics and lipidosis.

## Methods

The study population consisted of 16 RA patients (10 female; mean age 51 ± 13 years), 13 SLE patients (13 female; mean age 43 ± 9 years) and 12 age- and sex-matched controls (8 female; mean age 49 ± 15 years). Patients with previously known cardiovascular disease (CVD) were excluded. Participants underwent CMR at 1.5T and the assessments included cine, tagging, T1 mapping, T2-weighted, perfusion, late gadolinium (0.15mmol/kg gadoteric acid - Dotarem®) imaging and ECV quantification. Further assessments included proton (^1^H) and phosphorous (^31^P) MRS at 3T. Comorbid status, disease activity index and duration of disease were recorded for each subject.

## Results

Patients were well-matched with controls for sex, age and comorbidity (Table 1). The DAS28-CRP was 5 ± 1 in RA and the SLEDAI was 7 (4-11), respectively, indicating on-going disease activity. There were no differences in left ventricular (LV) size, mass and systolic function between RA and SLE patients when compared to controls (Table 2). Consistent with previously described findings, there were significant differences in strain, and tissue characteristics between the patients and controls. The PCr/ATP ratio was reduced in patients and measured 2.0 ± 0.3, 1.5 ± 0.3 and 1.4 ± 0.3 (p=0.02) in controls, RA and SLE, respectively. No differences were found in cardiac lipid content when RA and SLE patients were compared to controls. PCr/ATP ratio correlated with presence of LGE (R -0.38, p=0.14), MPRI (R 0.61, p<0.001), LA size (R -0.32, p=0.04), ECV (R -0.46, p=0.006) and volume fraction of T1 >990ms (R -0.73, p<0.001).

**Table 1:** Baseline characteristics

	Controls N=12	RA N=16	SLE N=13	P value
Female sex, n (%)	8 (67)	10 (63)	13 (100)	0.05

Age, years	49 ± 15	51 ± 13	43 ± 9	0.18

Hypertension, n (%)	0	3 (19)	2 (16)	-

Diabetes, n (%)	0	0	1 (8)	-

Hyperlipidaemia, n (%)	0	3 (19)	0	-

BMI, kg/m2	24 ± 3	25 ± 3	27 ± 5	0.12

Chloroquine, n (%)	N/A	11 (69)	11 (85)	-

Methotrexate, n (%)	N/A	15 (94)	1 (8)	-

Prednisolone, n (%)	N/A	2 (13)	9 (69)	-

Leflunomide, n (%)	N/A	3 (19)	1 (8)	-

Sulfasalazine, n (%)	N/A	3 (19)	0	-

Azathioprine, n (%)	N/A	0	4 (31)	-

Rituximab, n (%)	N/A	2 (13)	1 (8)	-

DAS28-CRP	N/A	5 ± 1	N/A	-

SLEDAI (median, IQR)	N/A	N/A	7 (4-11)	-

ESR, mm/hr (median, IQR)	N/A	7 (5-10)	10 (4-16)	-

CRP, mg/L (median, IQR)	1 (1-1)	5 (2-7)	4 (2-6)	<0.001

Duration of disease, years (median, IQR)	N/A	11 (7-14)	13 (6-16)	-

Duration of DMARDs, years (median, IQR)	N/A	7 (5-11)	8 (3-11)	-

Continuous data are mean ± SD unless otherwise indicated. Categorical data are frequency (percent) unless otherwise indicated. BMI, body mass index; CRP, C-reactive protein; DAS28-CRP (rheumatoid arthritis disease activity index incorporating 28 swollen and tender joint count as well as the C-reactive protein); ESR, erythrocyte sedimentation rate; IQR, interquartile range; RA, rheumatoid arthritis; SLE, systemic lupus erythematosus; SLEDAI, systemic lupus erythematosus disease activity index

**Table 2:** CMR and MRS findings

	Controls N=12	RA N=16	SLE N=13	P value
LVEDV indexed to BSA, ml/m2	78 ± 15	79 ± 23	74 ± 13	0.54

LVESV indexed to BSA, ml/m2	23 ± 5	23 ± 11	21 ± 6	0.75

LVEF, %	72 ± 4	72 ± 7	72 ± 5	0.96

LV Mass indexed to BSA, g/m2	53 ± 14	55 ± 11	47 ± 7	0.09

LA size, mm	27 ± 5	33 ± 6	29± 4	0.02

Mid SA circumferential strain	-18.7 ± 1.1	-17.5 ± 0.9	-16.5 ± 0.8	<0.001

Peak diastolic circumferential strain rate (s-1)	118 ± 21	88 ± 18	76 ± 24	<0.001

Presence of LGE (%)	0	8 (50)	3 (23)	-

Volume fraction of LGE>2SD (%)	0	3.4 ± 0.4	2.6 ± 0.3	-

Global myocardial T2 SI Ratio	1.5 ± 0.1	1.7 ± 0.3	1.9 ± 0.4	0.03

Volume fraction of oedema by T2 (%)	0	26 (18-39)	20 (13-31)	-

Average myocardial T1, ms	961 ± 12	968 ±25	987 ± 26	0.01

Volume fraction of T1>990ms (%)	3 (1-5)	34 (17-55)	41 (23-61)	<0.001

ECV (%)	26.5 ± 2.7	30.4 ± 3.1	32.2 ± 4.4	<0.001

MPRI	2.0 ± 0.3	1.6 ± 0.2	1.5 ± 0.5	<0.001

PCr/ATP	2.0 ± 0.3	1.5 ± 0.3	1.4 ± 0.3	<0.001

Cardiac lipid content (%)	0.49 ± 0.37	0.46 ± 0.33	0.58 ± 0.27	0.78

Hepatic lipid content (%)	1.70 ± 0.72	1.46 ± 0.58	2.17 ± 1.02	0.64

Continuous data are mean ± SD unless otherwise indicated. BSA, body surface area; ECV, extracellular volume; LA, left atrium; LGE, late gadolinium enhancement; LV, left ventricle/ventricular; LVEDV, left ventricular end-diastolic volume; LVEF, left ventricular ejection fraction; LVESV, left ventricular end-systolic volume; MPRI, myocardial perfusion reserve index; PCr/ATP, phosphocreatine to adenosine triphosphate ratio; RPP, rate pressure product; SA, short axis; SI, signal intensity, SLE, systemic lupus erythematosus

## Conclusions

In RA and SLE patients (with no overt cardiovascular disease) myocardial energetics are impaired, likely due to abnormal mitochondrial dysfunction. Abnormal myocardial energetics are associated with indices of myocardial fibrosis and oedema. There are no differences in myocardial lipid content between patients and controls.

## Funding

This study was funded by investigator-led grants from Guerbet and GlaxoSmithKline.

